# Development of sex- and genotype-specific behavioral phenotypes in a *Shank3* mouse model for neurodevelopmental disorders

**DOI:** 10.3389/fnbeh.2022.1051175

**Published:** 2023-01-09

**Authors:** Helen Friedericke Bauer, Jan Philipp Delling, Jürgen Bockmann, Tobias M. Boeckers, Michael Schön

**Affiliations:** ^1^Institute for Anatomy and Cell Biology, Ulm University, Ulm, Germany; ^2^International Graduate School in Molecular Medicine Ulm, Ulm University, Ulm, Germany; ^3^Deutsches Zentrum für Neurodegenerative Erkrankungen (DZNE), Ulm Site, Ulm, Germany

**Keywords:** Phelan-McDermid syndrome, *Shank3*, behavior, autism spectrum disorders, mouse model, muscular hypotonia, neurodevelopmental disorders

## Abstract

Individuals with a *SHANK3*-related neurodevelopmental disorder, also termed Phelan-McDermid syndrome or abbreviated as PMS, exhibit significant global developmental delay, language impairment, and muscular hypotonia. Also common are repetitive behaviors and altered social interactions, in line with a diagnosis of autism spectrum disorders. This study investigated the developmental aspect of autism-related behaviors and other phenotypes in a *Shank3*-transgenic mouse model. The animals underwent two sets of identical behavioral experiments, spanning motor skills, social and repetitive behavior, and cognition: baseline began at 5 weeks of age, corresponding to human adolescence, and the follow-up was initiated when aged 13 weeks, resembling early adulthood in humans. Interestingly, the animals displayed relatively stable phenotypes. Moreover, motor coordination and endurance were impaired, while muscle strength was unchanged. Surprisingly, the animals displayed only minor impairments in social behavior, but pronounced stereotypic and repetitive behaviors. Some behavioral tests indicated increased avoidance and anxiety. While spatial learning and memory were unchanged, knockout animals displayed slightly impaired cognitive flexibility. Female animals had similar abnormalities as males in the paradigms testing avoidance, anxiety, and cognition, but were less pathological in motor function and repetitive behavior. In all test paradigms, heterozygous *Shank3* knockout animals had either no abnormal or a milder phenotype. Accurate characterization of animal models for genetic diseases is a prerequisite for understanding the pathophysiology. This is subsequently the basis for finding suitable and, ideally, translational biomarkers for therapeutic approaches and, thereby reducing the number of animals needed for preclinical trials.

## 1. Introduction

### 1.1. *SHANK3* protein

*SHANK3* is a postsynaptic density protein in excitatory synapses encoded by the SH3 and multiple ankyrin repeat domains 3 gene (*Shank3*). It is mainly known to act as a structural protein directly or indirectly linking glutamate receptors with the actin cytoskeleton of a dendritic spine (Boeckers, 2006). However, *SHANK3*, with its multiple isoforms (Wang et al., 2014), appears to have other localizations and additional functions. Therefore, axonal, somatic, and nuclear expressions are described (Beri et al., 2007; Wang et al., 2014). *SHANK3* is also found in many non-neuronal tissues, including heart, liver, kidney, muscle, and myelinating cells (Lim et al., 1999; Lutz et al., 2020; Malara et al., 2022).

### 1.2. *SHANK3* deficiency in humans

Heterozygous *SHANK3* loss or *SHANK3* mutations in humans are thought to be the major cause for the neuronal developmental disorder frequently referred to as Phelan-McDermid syndrome (PMS). The most common cause is a microdeletion termed 22q13.3 deletion syndrome. As the name indicates, the deletion of the long arm of chromosome 22 leads to haploinsufficiency of various genes, including *SHANK3*. In most cases, affected individuals exhibit severe global developmental delay, intellectual disability, absent speech/severe speech delay, and muscular hypotonia. Furthermore, many patients display autism-related behaviors, as associated with a diagnosis of autism spectrum disorders (ASD), and have other comorbidities, such as sleep disturbances, epilepsies, hypoalgesia, dysmorphic features, and gastrointestinal dysfunction (Phelan and McDermid, 2011; Soorya et al., 2013; Sarasua et al., 2014; Levy et al., 2022; Nevado et al., 2022).

### 1.3. *SHANK3* deficiency in mice

To study neurodevelopmental disorders and ASD, and particularly PMS, several *Shank3* knockout (KO) mouse models were generated to investigate behavioral and molecular impacts of *Shank3* deletion (reviewed recently in Delling and Boeckers, 2021). With an extensive test battery in the domains of motor function, social and repetitive behavior, anxiety and avoidance behavior, and cognition, the *Shank3*Δ*11* mouse model, also denominated below as *Shank3* KO, was characterized in this study at two time points resembling periods in human adolescence and early adulthood (Dutta and Sengupta, 2016). Both females and males, as well as homozygous- and heterozygous- deleted animals, were equally examined and compared with wildtype (WT) animals.

## 2. Materials and methods

### 2.1. Animal ethics statement

*Shank3(–/–)* mice were previously described (Schmeisser et al., 2012). All mice were housed under standard laboratory conditions, with access to food and water *ad libitum*, and a 12 h dark/light cycle. WT, *Shank3*(+/–), and *Shank3*(–/–) KO mice used in this study were obtained from heterozygous breeding. The first set of behavioral experiments were conducted from postnatal week (PNW) 4 to 9, and the second behavioral set was performed on the same animals from PNW 13 to 18. Detailed information on the order of the performed tests, age at testing, and number of animals per group are summarized in Supplementary Table 1. All animal experiments were conducted in compliance with the guidelines for the welfare of experimental animals issued by the Federal Government of Germany, and approved by the Ethics Committee of Regierungspräsidium Tübingen, ID number: 1497.

### 2.2. Grid hanging test

Each mouse was placed on a horizontal grid and allowed to grasp the wire with all limbs. The grid was turned by 180°. The latency to fall was determined with a cutoff time of 180 s. The test was repeated (a maximum of three times) when the mouse fell within the first 3 s.

### 2.3. Grip strength test

Grip strength of all four limbs was simultaneously measured three times for each mouse, using a grip strength measurement device (BIO-GS3 from BIOSEB). The mouse was held at the tail and allowed to grab the metal grid of the device. After pulling the animal backward in the horizontal plane, the maximum force applied to the grid before the animal lost its grip was measured. Measurements were repeated three times per animal and mean values were used for further calculations.

### 2.4. Rotarod

The motor coordination and memory of the animals was assessed with the Rotarod test on five consecutive days, with three trials per day, and an inter-trial interval of 15–20 min. The mouse was placed on a Rotarod apparatus (Rota-Rod for Mouse ENV-575M Med Associated, Inc.) with an increasing speed from 4 to 40 rotations per minute over 5 min. The latency to fall off the rod, or when an animal was passively turning with the rod for two successive rotations, was determined.

### 2.5. Open field test

Animals were allowed to freely explore the open field arena (50 cm × 50 cm, with 12 lux in the center) for 30 min. This activity was tracked by video and analyzed with EthoVision 16 Software (Noldus Technologies). The total distance traveled was a measure for general locomotion. The entries in the border versus the central zone and the time spent in these two zones of the open field arena were recorded as well.

### 2.6. Barnes Maze and reverse Barnes Maze test

The Barnes Maze apparatus consists of a round platform 1 m in height and 1 m in diameter with 20 holes on the brim. On the first day, mice were allowed to explore the platform for 3 min to familiarize themselves with the setting without the bright lights on and without the escape box. The Barnes Maze test was performed on five consecutive days with two trials of 15–20 min each per day. The escape box was placed under one of the holes and remained in the same position for 5 days. The mice were allowed to explore the platform and to enter the escape box. During the test, the platform was brightly illuminated (approx. 600 lux). The maximum time to enter the escape box was set at 3 min. On the sixth day, the escape box was removed and replaced by a plastic cup. The mice were tracked for 3 min and the time spent in the target quadrant was measured. To assess cognitive flexibility of the mice, a reversal training phase was performed. The location of the escape box was changed from 120 to 180° and two trials of 15–20 min each were conducted per day during 3 days. On the fourth day, the reversal probe trial was performed as described above. The escape box and platform were cleaned with 60% ethanol and dried with paper tissues between each trial. Automated video tracking was performed with EthoVision 16 Software (Noldus Technologies).

### 2.7. Direct social dyadic test

Both male and female mice were housed individually 3 days before the test day. The test mouse was placed in a fresh test cage filled with bedding material in a soundproof room. After 20–25 min of habituation, a stranger C57BL/6 mouse of the same sex and age was introduced. The free interaction of the animals was videotaped for 5 min (Panasonic HC-V160 video camera). Analysis of the time the two animals spent in close proximity (< 3.5 cm) was performed with the EthoVision 16 Software (Noldus Technologies).

### 2.8. Self-directed behavior

The test mouse was placed in a fresh standard cage with bedding material in a soundproof room. After 10 min habituation to the cage, the animal’s behavior was recorded for 10 min. The time spent self-grooming and digging, as well as the number of self-grooming and digging bouts, was measured manually.

### 2.9. Nestlet shredding test

One week before the test, animals received cotton nestlets for nesting to become accustomed to the new material. Mice were kept individually in a new home cage. One hour before the dark phase, a weighed single cotton nestlet was placed in the cage. The following morning, unprocessed nesting material was collected and dried overnight. The intact nestlet was weighed to calculate percentage of shredded nestlet.

### 2.10. Marble burying test

The marble burying test was performed in a soundproof room. After habituation to the room for 30 min, the test mouse was placed in a clean standard cage containing a 5 cm thick layer of fresh bedding material, with 18 glass marbles placed in a regular pattern on the bedding surface. The cage was closed with a filter top lid without a grid to avoid climbing of the animal. After 30 min, the number of marbles buried in the bedding, up to two-thirds in depth, was counted.

### 2.11. Statistical analysis

Data are displayed as mean ± standard error of the mean (SEM). Statistical analysis was performed, and graphs were generated using GraphPad Prism 8 and/or R Studio 3 Software. Normal distribution was tested by Shapiro-Wilk test. Data were analyzed by two-way analysis of variance (2-way ANOVA) followed by Tukey’s multiple comparison test with genotype and sex as factors. Because repeated measures ANOVA cannot handle missing values in GraphPad Prism 8.0, the data with missing values were analyzed by fitting a mixed model using a compound symmetry covariance matrix, and were fitted using Restricted Maximum Likelihood (REML). The results of the mixed model can be interpreted as repeated measures ANOVA. For categorical data, Fisher’s exact test was used. Intraindividual animal comparison between adolescent and adult age was analyzed by paired two-tailed *t*-test for parametric data and by Wilcoxon matched pairs signed rank test for non-parametric data. Significance levels (*p*-values) were set to 0.05 (*p* ≤ 0.05*, *p* ≤ 0.01^**^, *p* ≤ 0.001^***^, *p* ≤ 0.0001^****^) with 95% confidence interval. The animals included were female *Shank3*(+/+) *n* = 10, female *Shank3*(+/–) *n* = 11, female *Shank3*(–/–) *n* = 9, male *Shank3*(+/+) *n* = 11, male *Shank3*(+/–) *n* = 10, and male *Shank3*(–/–) *n* = 9. Detailed statistical information for all data sets can be found in the Supplementary material.

## 3. Results

In the following section, the phenotypic characteristics of the *Shank3*Δ*11* mouse model in the domains of motor function, repetitive and social behavior, anxiety and avoidance behavior, and cognition are reported. The study was conducted longitudinally with two identical sets of behavior tests performed at two different ages: from PNW 4–9 and PNW 13–18. These intervals span the time corresponding to human adolescence and early adulthood, respectively (Dutta and Sengupta, 2016). Both males and females were analyzed, and all three genotypes were compared to each other.

### 3.1. *Shank3* KO mice display impairment in motor coordination and endurance

One of the first visible symptoms of PMS is neonatal muscular hypotonia. So-called “floppy infants” have feeding problems due to difficulties in sucking and swallowing. Hypotonia frequently persists in PMS patients throughout life, leading to gait instability, problems in gross and fine motor coordination, and muscle weakness (Phelan and McDermid, 2011; Soorya et al., 2013; Sarasua et al., 2014). Therefore, this study focused on motor function, both in early life and during development of the *Shank3* KO mouse model.

**FIGURE 1:**
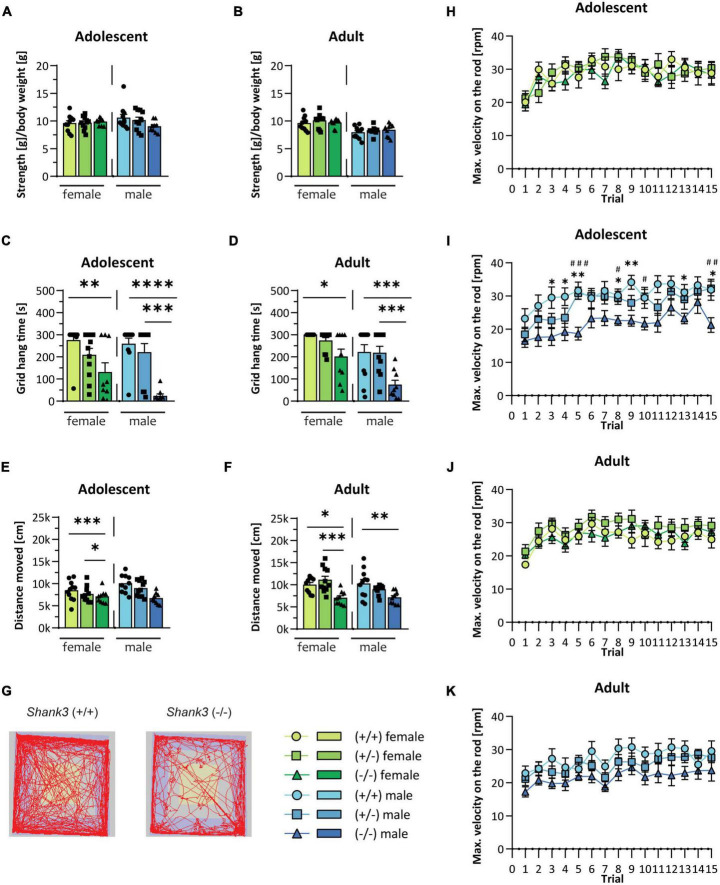
Motor function in *Shank3-*transgenic mice. Muscle function is analyzed in male and female wildtype, *Shank3*(+/–), and *Shank3*(–/–) mice at adolescent and adult age by determining **(A,B)** the muscle strength and **(C,D)** the grid hang time in the grid hanging test. **(E,F)** General locomotion was measured as the distance moved during a 30 min trial in the open field test. **(G)** Representative tracking images of the open field test from a wildtype and a *Shank3*(–/–) mouse are displayed. Motor memory and coordination were tested in three trials per day on five consecutive days on the accelerating Rotarod (4 to 40 rotations per minute within 5 min). **(H–K)** The maximal velocity, when the animal fell from the rod, is displayed. Asterisks (*) indicate significant differences between wildtype and *Shank3*(–/–) mice; # indicates significant differences between *Shank3*(+/–) and *Shank3*(–/–) mice. All data are represented as means with SEM with 9–11 animals per group. Significance levels (*p*-values) are set to 0.05 (*p* ≤ 0.05^*/#^, *p* ≤ 0.01^**/##^, *p* ≤ 0.001^***/###^, *p* ≤ 0.0001^****^) with 95% confidence interval. See Supplementary Table 2 for detailed statistical information.

First, the righting reflex during pup development over postnatal days 2 to 10 was analyzed. There were no significant differences found in the latency to turn from the back to all four paws between the genotypes (see Supplementary Figures 1A, B) or in body weight (Supplementary Figures 1C, D), and also later in development (Supplementary Figures 1E, F). Later during development, *Shank3* KO animals displayed strong but selective impairments in their motor function. The grip strength of all four limbs was not altered in the grip strength test (Figures 1A, B). However, the animals displayed distinct motor dysfunctions with reduced endurance and coordination when several muscle groups were required to coordinate or be activated for a longer period, such as during hanging and climbing at a grid in the grid hanging test (Figures 1C, D) or during the Rotarod test (Figures 1H–K). Furthermore, a general reduction of locomotion in the open field arena (Figures 1E–G) was detected. Motor impairments, particularly the performance on the Rotarod, were more pronounced at baseline and in male *Shank3* KO animals. Heterozygous animals were uniformly unaffected in these tests. The motoric function in females homozygous *Shank3* KO mice was less impaired as compared to male homozygous KO animals.

### 3.2. Autism-like behavior in *Shank3* KO mice

**FIGURE 2:**
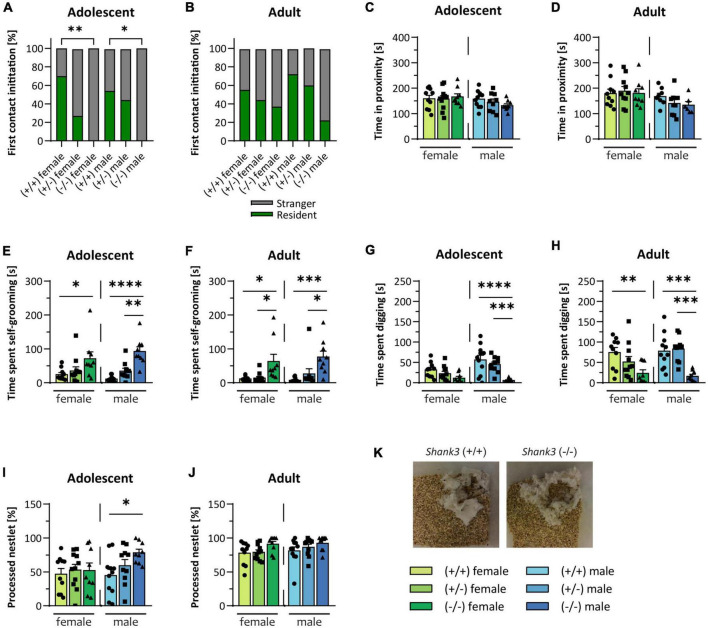
Autism-related behaviors in *Shank3*-transgenic mice. Social interest of *Shank3*(+/–) and *Shank3*(–/–) compared to wildtype was assessed in the direct social dyadic test, in which the testing animal was able to directly interact with a sex-and age-matched stranger conspecific. **(A,B)** Shows whether the first contact in the direct social dyadic test was initiated by the testing mouse or the stranger mouse. **(C,D)** The time the two animals spent in close proximity to each other in the direct social dyadic test are determined. Different behaviors **(E–H)** were recorded within a 10 min observation session (after 10 min of habituation to a new cage). **(E,F)** The time spent self-grooming and **(G,H)** the digging time (moving the cage bedding with front and/or hind paws) were analyzed. **(I,J)** In the nest building test, the amount of shredded cotton nestlet was determined. **(K)** Representative images of processed nestlets from a wildtype (left) and a *Shank3*(–/–) (right) mouse are displayed. All data are represented as means with SEM with 9–11 animals per group. Significance levels (*p*–values) are set to 0.05 (*p* ≤ 0.05*, *p* ≤ 0.01^**^, *p* ≤ 0.001^***^, *p* ≤ 0.0001^****^) with 95% confidence interval. See Supplementary Table 3 for detailed statistical information.

The two key features of ASD are repetitive/restricted behavior, and impairments in social interaction and communication (American Psychiatric Association, 2013). In the direct social dyadic test, the tested mouse was allowed to freely interact with an age-matched stranger mouse of the same sex. Interestingly, the *Shank3* KO mice frequently froze when the stranger mouse was introduced into the cage, and the first contact at baseline experiments was always initiated by the stranger mouse, but not in the follow-up experiment. In contrast, the WT mice approached the stranger mouse at baseline in an equal frequency as compared to the stranger mouse (Figures 2A, B). After the first contact, *Shank3* KO mice spent the same amount of time in close proximity to the stranger mouse in comparison to the WT mice (Figures 2C, D). Young male animals displayed a slightly decreased ultrasonic vocalization call rate during the social dyadic test (see Supplementary Figures 1G, H). Sociability of *Shank3* KO animals was assessed in the three-chamber sociability test, in which the testing mouse can freely decide to spend time in the chamber with a stranger conspecific or in a chamber with an unfamiliar, novel object. Heterozygous, as well as homozygous *Shank3* KO animals, were sociable at both ages. They preferred to spend more time in the chamber with the living animal compared to the inanimate stimulus (see Supplementary Figures 1I, J). Another core symptom of ASD is repetitive and restricted behavior. *Shank3* KO mice spent increased time on self-grooming (Figures 2E, F), and, in agreement with repetitive behavior, significantly reduced time digging. This was observed especially in males at both ages (Figures 2G, H). In addition to increased self-grooming, the nestlet shredding test can be used as another readout for repetitive, compulsive-like behavior in mice (Angoa-Pérez et al., 2013). Increased nestlet shredding was observed in male *Shank3* KO mice at baseline (Figure 2I), but not in females or in the follow-up testing (Figures 2J, K). Heterozygous animals uniformly displayed no abnormalities, but showed trends toward the full KO genotype in some test paradigms, such as self-grooming and marble burying. In summary, in most autism-related tests, results were similar for both female and male animals.

### 3.3. Assessment of anxiety, spatial memory, and cognitive function in *Shank3* KO mice

**FIGURE 3:**
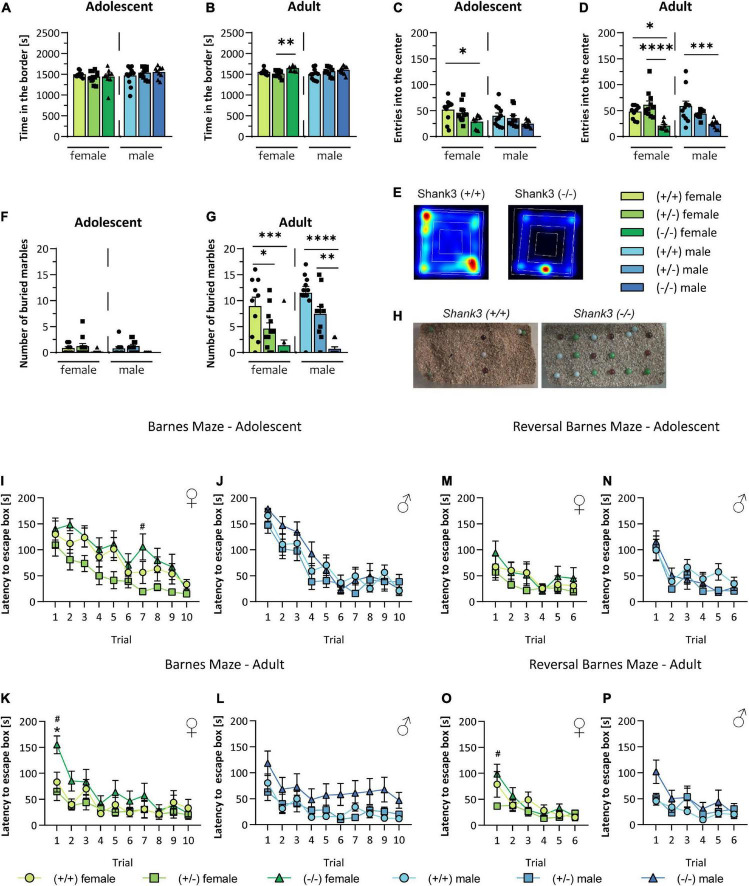
Assessment of anxiety, cognitive function, and spatial learning and memory in *Shank3*-transgenic mice. Anxiety of *Shank3*(+/–) and *Shank3*(–/–) mice compared to wildtype mice was determined in the open field test. Thereby, **(A,B)** the time spent in the border zone and **(C,D)** the number of entries into the center zone of the open field arena were determined. **(E)** Representative heat maps of the 30 min open field test of wildtype and *Shank3*(–/–) mice are displayed. **(F,G)** Number of buried marbles and **(H)** representative images of buried marbles in the marble burying test are shown. In the Barnes Maze test, the spatial orientation and memory of the animals was tested. **(I–L)** The latency to enter the escape box in two trials per day on five consecutive days was analyzed, while the escape box stayed in the same position. Subsequently, the reversal Barnes Maze test was performed to determine the cognitive flexibility of the animals. Here, the position of the escape box was changed (120 to 180°) and **(M–P)** the latency to enter the escape box in two trials per day on three consecutive days was determined. Asterisks (*) indicate significant differences between wildtype and *Shank3*(–/–) mice; # indicates significant differences between *Shank3*(+/–) and *Shank3*(–/–) mice. All data are represented as means with SEM with 9–11 animals per group. Significance levels (*p*-values) are set to 0.05 (*p* ≤ 0.05^*/#^, *p* ≤ 0.01^**^, *p* ≤ 0.001^***^, *p* ≤ 0.0001^****^) with 95% confidence interval. See Supplementary Table 4 for detailed statistical information.

Anxiety-like behavior can be measured in the open field test and becomes apparent when the animal spends more time in the outer zone of an arena and avoids entering the brighter center zone. Here, the time the animals spent in the outer zone was unchanged in young mice (Figure 3A), but minimally increased in the older female *Shank3* KO mice (Figure 3B). Furthermore, young male KO animals entered the center area of the arena less frequently (Figure 3C), as did adult animals of both sexes (Figure 3D; heatmap is displayed in Figure 3E). Animals at baseline did not bury marbles in the marble burying test. While older WT animals learnt this independent of genotype, KO animals of both sexes almost invariably did not bury or move marbles at follow-up. Results for heterozygous animals were situated between the other genotypes, but not significantly altered (Figures 3F, G; exemplary images in Figure 3H). The Barnes Maze test measures spatial learning and memory in mice. By adding a reversal Barnes Maze test to the protocol, the cognitive flexibility of the animals can be analyzed [for a detailed review, see Gawel et al. (2019)]. Adolescent *Shank3* KO mice of both sexes displayed no differences in spatial learning (Figures 3I, J) or in cognitive flexibility (Figures 3M, N). Adult female *Shank3* KO mice required significantly longer to enter the escape box on the first trial compared to heterozygous or WT animals, but memorized the new location in subsequent trials (Figure 3K). For all trials, adult male *Shank3* KO mice needed slightly onger, but not significant, to enter the escape box compared to both the other genotypes (Figure 3L). In the first trial of the reversal Barnes Maze test, in which the location of the escape box was changed, both male and female mice were able to reorientate themselves and to memorize the new location of the escape box, but an insignificant trend of increased latency was observed in adult homozygous KO mice (Figures 3O, P).

### 3.4. Intraindividual performance at baseline and follow-up

**FIGURE 4:**
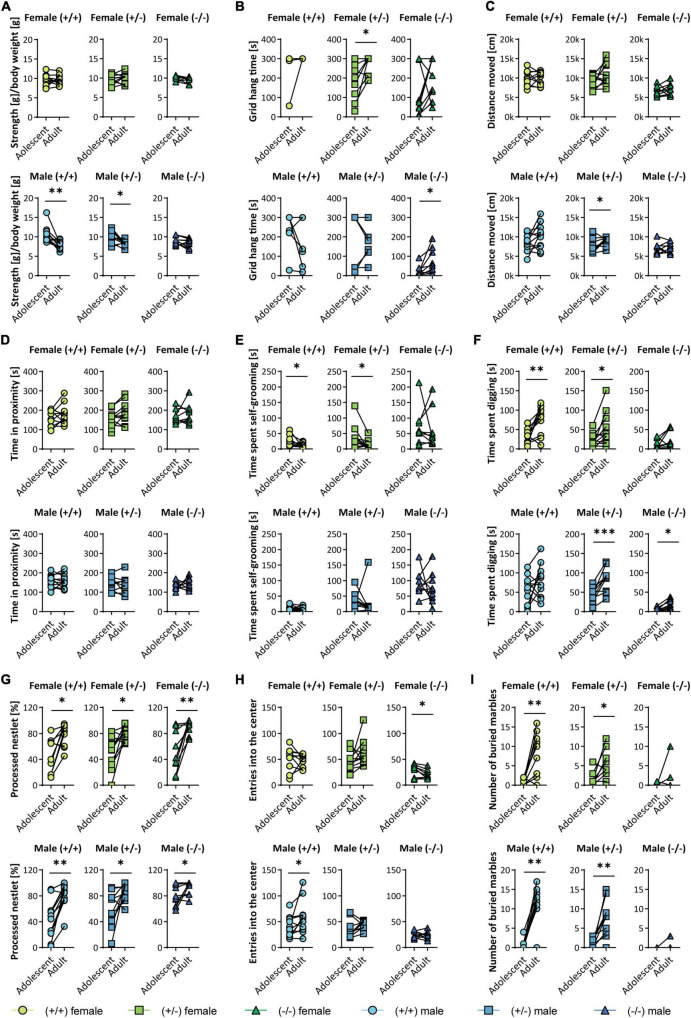
Trajectory analysis of individual animals between baseline (adolescent) and follow-up (adult) *Shank3*(+/–). A selected set of tests is analyzed longitudinally and displayed as follows: female (upper panels) and male (lower panels), wildtype (left), *Shank3*(±) (middle), and *Shank3*(–/–) (right) mice. Graphs are displayed for **(A)** the normalized strength [strength/body weight] in the grip strength test for all four paws, **(B)** grid hang time in the grid hanging test, **(C)** the distance moved in the open field test, **(D)** the time the testing mouse spent in proximity (< 3.5 cm) to a same-sex stranger in the direct social dyadic test, **(E)** the time the animal spent self-grooming and **(F)** digging, **(G)** the percentage of processed material in the nestlet shredding test, **(H)** the number of entries into the center zone of the open field arena during a 30 min trial, and **(I)** the number of buried marbles in the marble burying test. Data are presented for each individual animal at baseline and follow-up and was analyzed by paired two-tailed *t*-test for parametric data and by Wilcoxon matched pairs signed rank test for non-parametric data. Significance levels (*p*-values) are set to 0.05 (*p* ≤ 0.05*, *p* ≤ 0.01^**^, *p* ≤ 0.001^***^) with 95% confidence interval. Detailed statistical information for all data sets can be found in Supplementary Table 5.

Intraindividual comparison of the results was performed for some tests of the two identical experimental series at baseline and follow-up (Figure 4). In the grip strength test (Figure 4A), the results of the strength per body weight were stable over time, with only adult male WT and heterozygous KO mice displaying decreased strength. The grid hang time (Figure 4B) was highly variable in all groups at both time points, but generally very low in homozygous male *Shank3* KO. There was a minor improvement in heterozygous females and homozygous *Shank3* KO males. The distance traveled in the open field test was largely stable (Figure 4C), as was the time animals spent in close contact with a stranger mouse (Figure 4D). Self-grooming was more variable in homozygous *Shank3* KO animals of both sexes (Figure 4E). Digging increased significantly over time in most genotypes (Figure 4F). In all groups, animals shredded nestlets more at follow-up, with male homozygous *Shank3* KO animals already having a high level at baseline (Figure 4G). While, in the open field test, entries into the center were highly variable in all WT and heterozygous groups at both time points, they were much more homogeneous and less frequently observed in both male and female homozygous animals (Figure 4H). All animals scarcely buried marbles at baseline, but while WT and heterozygous animals did so later, this was not seen in homozygous *Shank3* KO animals of both sexes (Figure 4I).

## 4. Discussion

### 4.1. Summary

Abnormalities were found in many behavioral domains that are similarly observed in PMS. In motor function, the KO animals displayed intact absolute muscle strength, but severely impaired motor coordination and endurance of several muscle groups, as well as general hypoactivity. The *Shank3* KO mice displayed only minor differences in social behavior, with only the first contact initiation with a stranger mouse being impaired. In addition to social interaction impairments, ASD is characterized by repetitive behavior, which was present in male and female KO mice at both ages. Furthermore, animals of both sexes had increased fear and presented avoidance behavior toward inanimate objects and in initial contact with a novel conspecific. In the Barnes Maze test, only adult female KO mice displayed a disability in the first test round, while a tendency for reduced spatial learning was observed in adult KO males. Cognitive flexibility was not significantly reduced. In conclusion, homozygous female and male KO animals had comparable phenotypes, with females performing better only in some tests in the motor domain. Heterozygous animals frequently showed no or only trend-like changes and were, therefore, situated between WT and full KO phenotypes, in some motor and repetitive behavior tests. Most changes in homo- and heterozygous KO mice were stable, and partially more pronounced at either baseline or follow-up.

### 4.2. Comparison with previous *Shank3*-related mouse studies

The behavior of different isoform-specific *Shank3* transgenic mouse lines was comprehensively summarized by Delling and Boeckers in 2021. They distinguished between the domains “Behavior” (Social, Stereotypes, and other Behavior, such as anxiety, avoidance, etc.) and “Neurology” (Cognition, Motor, Sensory, etc.). A similar classification was applied in the assessment of the results in comparison to other studies. *Shank3* transgenic rodents have been extensively studied in many previous analyses. Different KO strategies were used, such as isoform-specific versus full KO, and brain region-or development-dependent versus constitutive. Certain phenotypes appeared to be associated with these aspects. Studies with female and heterozygous animals, and with a baseline and a follow-up examination, as in the present study, are very rare (Delling and Boeckers, 2021). The *Shank3*Δ*11*-specific transgenic mouse was tested in several previous studies (Vicidomini et al., 2017; de Chaumont et al., 2019; Ponzoni et al., 2019; Lutz et al., 2020; Urrutia-Ruiz et al., 2022). The most comprehensive study was performed by Vicidomini et al. Some tests comparable to those in the present study were performed, but both studies also performed individual tests. Some experimental procedures showed similar results, but others differed. While the same mouse line in Vicidomini et al. displayed reduced social motivation, interaction, and recognition in the “Behavior” section, this phenotype was only slightly pronounced in the present study. *Shank3* KO animals displayed significantly reduced initial contact with a stranger animal in the direct social dyadic test, but were unremarkable in other social paradigms, unlike observed by Vicidomini et al. They used different protocols, as well as the DBA/2 mouse strain as the stranger animal in the three-chamber sociability test. In the present study, C57BL/6 mice were used as stranger mice, which might have led to different reactions of the animals toward the social stimulus. These two differences might explain the discrepancy between the two studies regarding social interaction impairments. Similar to the present study, they found highly repetitive and avoidant behavior. Therefore, the reduced initial contact in the direct social dyadic test can also be interpreted as avoidance of initial social contact. Of interest were the differences in the domain “Neurology”. Cognitive flexibility was only altered in adult female KO mice in the present study, but the animals in Vicidomini et al. displayed a stronger pathological phenotype. In this study, cognitive flexibility was assessed with the Barnes Maze test, while in the Vicidomini et al. study, it was assessed by the Water Morries maze test, which is strongly dependent on motor function and anxiety levels of the animals (Vicidomini et al., 2017). The increased anxiety observed in the KO animals during the open field test in the present study was also observed in the same mouse model in the elevated-plus maze (Urrutia-Ruiz et al., 2022). The early life neuromotor development of another *Shank3* KO mouse model was recently studied. The pups were slower in a negative geotaxis test and opened their eyes later than WT litter mates. Comparable to the findings presented here, there were also no differences in the righting reflex (Pillerová et al., 2022). Motor phenotypes in this study were largely consistent with previous studies, showing reduced mobility and coordination of muscle groups (de Chaumont et al., 2019; Lutz et al., 2020). As was observed in a previous study with the same mouse line as in the present study, the heterozygous transgenic mice were not significantly different from WT in all test paradigms (Vicidomini et al., 2017).

Females resembled SHANK3-deficient males in most test paradigms. However, in some tests, they had less pronounced phenotypes, particularly in those indicating motor deficits. This is compatible with MRI (magnetic resonance imaging) abnormalities in the same mouse line, which were also more pronounced in males, particularly in areas involved in motoric functions, such as the globus pallidus and thalamus (Schoen et al., 2019).

In the following section, the motor phenotypes, stereotypies, and avoidant behavior are compared in terms of possible brain regions involved. The strongest phenotypes in the present study were motor deficits, stereotypies, and avoidant behavior. Motor centers of the brain include the motor cortex, basal ganglia, thalamus, and cerebellum. These are all areas that strongly express SHANK3 in an isoform-specific manner (Peça et al., 2011; Wang et al., 2014) in both mice and human brains (Wan et al., 2021). In a previous study, grooming was shown to occur in another isoform-specific KO of *Shank3*, at a stage corresponding to early childhood. Motor phenotypes are already present at postnatal day 15 and persist into adulthood (Peixoto et al., 2019). Reduced digging bouts and increased self-grooming, as signs for repetitive behavior, were observed in a *Shank2* KO mouse model (Schmeisser et al., 2012). In a previous MRI study, *Shank3* KO mice from the same mouse line as in the present study were also studied longitudinally. Several brain regions were volumetrically altered. While the hippocampus and thalamus were reduced in size, the cerebellum, striatum, and globus pallidus were enlarged (Schoen et al., 2019). Kim and colleagues suggested a central role for the cortico-basal ganglia-thalamic circuits in repetitive behavior in autism mouse models and also discussed the role of SHANK3. SHANK3, unlike SHANK2, is highly expressed in the striatum and cerebellum. *Shank3* KO mice display a more pronounced self-grooming phenotype, whereas *Shank2* KO mice are more hyperactive. Marble burying is difficult to classify because, on the one hand, it is increased in some ASD models, and, on the other hand, when decreased, as observed in the present study, can also indicate avoidance and anxiety (Kim et al., 2016). The strong phenotype of avoiding inanimate objects, as observed, for example, in the marble burying test or the novelty preference task, has previously been reported and discussed in other transgenic *Shank3* mouse models (Kouser et al., 2013; Lee et al., 2015; Speed et al., 2015; Bidinosti et al., 2016; Jaramillo et al., 2016; Wang et al., 2016; Drapeau et al., 2018; Kabitzke et al., 2018; Guo et al., 2019). The role of the striatum in repetitive behavior in autism mouse models is exemplified in two previous studies (Peça et al., 2011; Bey et al., 2018). In one of the first characterization studies of an isoform-specific *Shank3* KO, presumably affecting more isoforms than in the study conducted here, a highly repetitive behavior was also conspicuous. The striatum of the KO animals was enlarged and the mice were also anxious. The morphology and function of the neurons was also severely disrupted, possibly contributing to the phenotype (Peça et al., 2011). In the second study mentioned, different isoform-and brain region-specific *Shank3* KO mouse lines were generated, including KO in forebrain neurons and striatal neurons. Silencing of the gene in the forebrain was sufficient to induce self-grooming. Interestingly, global KO animals were hypermobile in the open field test, whereas forebrain-specific KO animals and those in which *Shank3* was knocked out in striatal medium spiny neurons of the indirect pathway were hyperactive. This suggests distinct roles of *SHANK3* in different brain regions (Bey et al., 2018).

In this section, the stability of phenotypes between baseline and follow-up observed in this study is discussed. Overall, few studies have been conducted longitudinally. In previous studies of the same mouse line examined in the present research, animals were also examined at two time points similar to the present baseline and follow-up. While brain morphological changes were stable with age (Schoen et al., 2019), changes in fractional anisotropy (intactness of axonal tracts) were strikingly altered only in the younger animals (Jesse et al., 2020).

### 4.3. Limitations

It is possible that learning effects influenced the results of the second set of experiments because the animals in the follow-up experimental series had previously performed the tests. However, because the phenotypes were still robustly visible later, we assumed a particular rigidity of the pathological characteristics of the transgenic animals can be assumed.

In some tests, a higher number of animals would have probably revealed more significant pathological changes in the transgenic animals.

Additionally, the homozygous *Shank3* transgenic animals still expressed isoforms and, therefore, corresponded more to a haploinsufficient situation similar to PMS.

### 4.4. Translational comparison to Phelan-McDermid syndrome

In this section, results of this study are compared translationally with what is known about the phenotype in male and female individuals with *SHANK3* deficiency and with the natural history in PMS, and only partially with ASD. The pathophysiology of ASD is highly heterogeneous and still poorly understood; moreover, not all PMS individuals have autism. Unlike PMS, there is a strong predominance of males affected with ASD (Lord et al., 2020). The here presented our data also confirmed this. Females differed only slightly from males. Social interaction was not affected in most animals, except for a pronounced avoidance at first contact. Therefore, a strong face validity of our mouse model can be postulated. Moreover, beyond a model for shankopathies, the isoform-specific *Shank3* mouse model presented corresponds better in generic terms to a model for neurodevelopmental disorders with partly autistic behavior, which is mainly expressed by strong repetitive behavior. Most phenotyping studies of PMS have been conducted in children, so knowledge is limited in adolescents and adults who match the age of the animals studied here. It must be considered that PMS is associated with haploinsufficiency of *SHANK3*. However, the heterozygous animals in the present study displayed very few abnormalities. The fact that heterozygous animals performed better than homozygous transgenic animals can be explained by the gene dosage. Moreover, isoforms of *SHANK3* are still present in homozygous KO mice. Zinc in the diet of the mice might also mitigate some of the phenotypes in heterozygous and homozygous *Shank3* KO mice, because *SHANK3* clustering at synapses is zinc-mediated (Grabrucker et al., 2011). Mouse lines with more deleted isoforms of *Shank3* partly show more distinct phenotypes (Delling and Boeckers, 2021).

In the present study, males and females had a similar phenotype with a general stability between baseline and follow-up tests. In some test paradigms, homozygous KO animals displayed amelioration, while deterioration was detected in others. Only in motor tests were females less conspicuous. Social interaction was hardly affected in both sexes. The natural history of PMS is still poorly understood. There are some studies on intersex comparisons and anecdotal reports on the course of certain phenotypes. As suggested by the results of the present study, the motor phenotype in PMS is very complex and includes, apart from hypotonia, gait and posture abnormalities (Kolevzon et al., 2014). Muscular hypotonia is particularly severe in infancy and often improves with age (Phelan and McDermid, 2011; Soorya et al., 2013; Levy et al., 2022). In one of the largest phenotyping studies, Sarasua and colleagues showed that hypotonia decreases slightly with age (Sarasua et al., 2014). However, in the age groups corresponding to the animals in the present study, it remained stable and affected > 50% of all individuals in both age groups at baseline and follow-up. While some symptoms, such as problematic behavior, improve with age, ASD and some motor phenotypes become more severe. This was observed in cases that matched in the comparison of the age groups corresponding to those in the present study. Many phenotypes in PMS individuals were age-specific but relatively stable overall. However, the number of cases was too small for a more in-depth statement in this cohort study. In more than 50 individuals, the development could be followed *via* follow-up studies. Many of the phenotypic characteristics remained stable, while some displayed improvement. Phenotypic differences between the sexes were barely reported (Sarasua et al., 2014). Another cohort study also showed no clear differences between male and female individuals (Nevado et al., 2022). In summary, the model in the present study displayed an age-robust phenotype, as do individuals with PMS. Unlike PMS, female animals with *SHANK3* deficiency were less affected in the motor domain. However, it may also be that differences between the sexes have not yet been detected because of a lack of research on individuals with PMS that targeted testing of specific motor functions, such as differentiating between strength and endurance or coordination. The remaining isoforms could also have had a significant impact on some of the paradigms. The *Shank3* KO animals in the present study displayed a very robust phenotype in the motor domain, but significantly more interindividual differences on tests of autism-like behavior. This is consistent with descriptions in large phenotyping studies with commonly seen hypotonia, but variable social or behavioral abnormalities in PMS (Phelan and McDermid, 2011; Sarasua et al., 2014; Nevado et al., 2022).

The intraindividual comparison between baseline and follow-up yielded very interesting results. Some trends emerged that are also observed in PMS individuals. Some symptoms, such as self-grooming, were highly variable in their respective ages and over time in the homozygous *Shank3* KO animals. Accordingly, only a proportion of individuals with PMS are affected by ASD (Phelan and McDermid, 2011; Sarasua et al., 2014). This variability may also explain the reports on regression observed in some individuals with PMS (Phelan and McDermid, 2011; Levy et al., 2022). The distinct motor phenotype in the grid hanging test showed improvement over time in male homozygous *Shank3* KO animals. In humans with PMS, there are also reports of a reduction in hypotonia with increasing age (Phelan and McDermid, 2011; Soorya et al., 2013; Levy et al., 2022). Some phenotypes were very stable, such as avoidance of the center zone in the open field test, as were many symptoms also seen in PMS at different ages (Sarasua et al., 2014).

### 4.5. Outlook

The accurate characterization of a transgenic animal disease model is important to demonstrate its face validity and to elucidate pathomechanisms, for both sexes and over development. Only then can observed phenotypes be translated to a human disease condition. Such phenotyping studies may also help to identify translational readouts for prospective pharmaceutical rescue and preclinical studies.

## Data availability statement

The original contributions presented in this study are included in the article/Supplementary material, further inquiries can be directed to the corresponding authors.

## Ethics statement

The animal study was reviewed and approved by Regierungspraesidium Tübingen and the local Ethics Committee at Ulm University; ID number: 1497.

## Author contributions

HB, TB, and MS designed the project and experiments. JB supervised animal breeding. HB performed the experiments, analyzed the data, and created the figures with the supervision of TB and MS. JD assisted with the data analysis. HB and MS jointly wrote the manuscript. All authors contributed to the article and approved the submitted version.
