# Supplementary material for: Development of sex- and genotype-specific behavioral phenotypes in a Shank3 mouse model for neurodevelopmental disorders
Source: Front Behav Neurosci. 2023 Jan 9;16:1051175. doi: 10.3389/fnbeh.2022.1051175 (PMC9868822; doi:10.3389/fnbeh.2022.1051175)
Supplement: Supplementary file 1 [file Data_Sheet_1.pdf]

Supplementary Material

1 Supplementary Methods

1.1 Righting reflex

To study the righting reflex of the pups, births were controlled each morning and evening. Pups of both sexes were individually identified with long-lasting sub-cutaneous tattoos (green tattoo paste, Ketchum Manufacturing Inc.) on the paws on postnatal day 1 (PND1). On PND 2, 4, 6, 8, and 10, the righting reflex and the body weight of the pups were determined. The pups were placed on a flat surface on their back and the time till they turned into a normal position was determined. The maximum latency time to right themselves up was set to 120 s.

1.2 Ultrasonic vocalization (USV) recording during direct social dyadic test

During the direct social dyadic test, the USV of the mice were recorded with a Condenser ultrasound microphone Polaroid/CMPA, the interface UltraSoundGate 116H/85, and an Avisoft SASLab Pro Recorder (Avisoft Bioacustics; sampling frequency 300 kHz; FFT-length: 1024 points; 16-bit format). USV was analyzed with the open source MATLAB-based Vocal Mat software developed and described by Fonseca and colleagues (Fonseca et al., 2021) to determine the call rate.

1.3 Three-chamber sociability test

To measure sociability in the animals, the three-chamber sociability test was applied. The mouse was placed in the center of a three-chambered plexiglass apparatus and allowed to explore all three chambers with one empty cage to both the left and right for 10 min. In a second phase of the test, two similar objects were placed in the cages, and the mouse was allowed to freely explore the three-chamber apparatus for another 10 min. In the third phase, the sociability test was performed. Therefore, one stranger C56/BL6 mouse of the same sex and age was placed in one cage and a novel object was placed in the other cage. Again, the mouse was allowed to explore the set-up for 10 min. All test phases were video-recorded and tracked with EthoVision 16 software (Noldus Technology). Animals that spent most of the test time climbing on the cages to escape the three-chamber apparatus were considered not to be attending the test and were excluded from the data.

2 Supplementary References

Fonseca, A.H.O., Santana, G.M., Bosque Ortiz, G.M., Bampi, S., Dietrich, M.O., 2021. Analysis of ultrasonic vocalizations from mice using computer vision and machine learning. eLife 10, 1–22. https://doi.org/10.7554/eLife.59161

3 Supplementary Figures

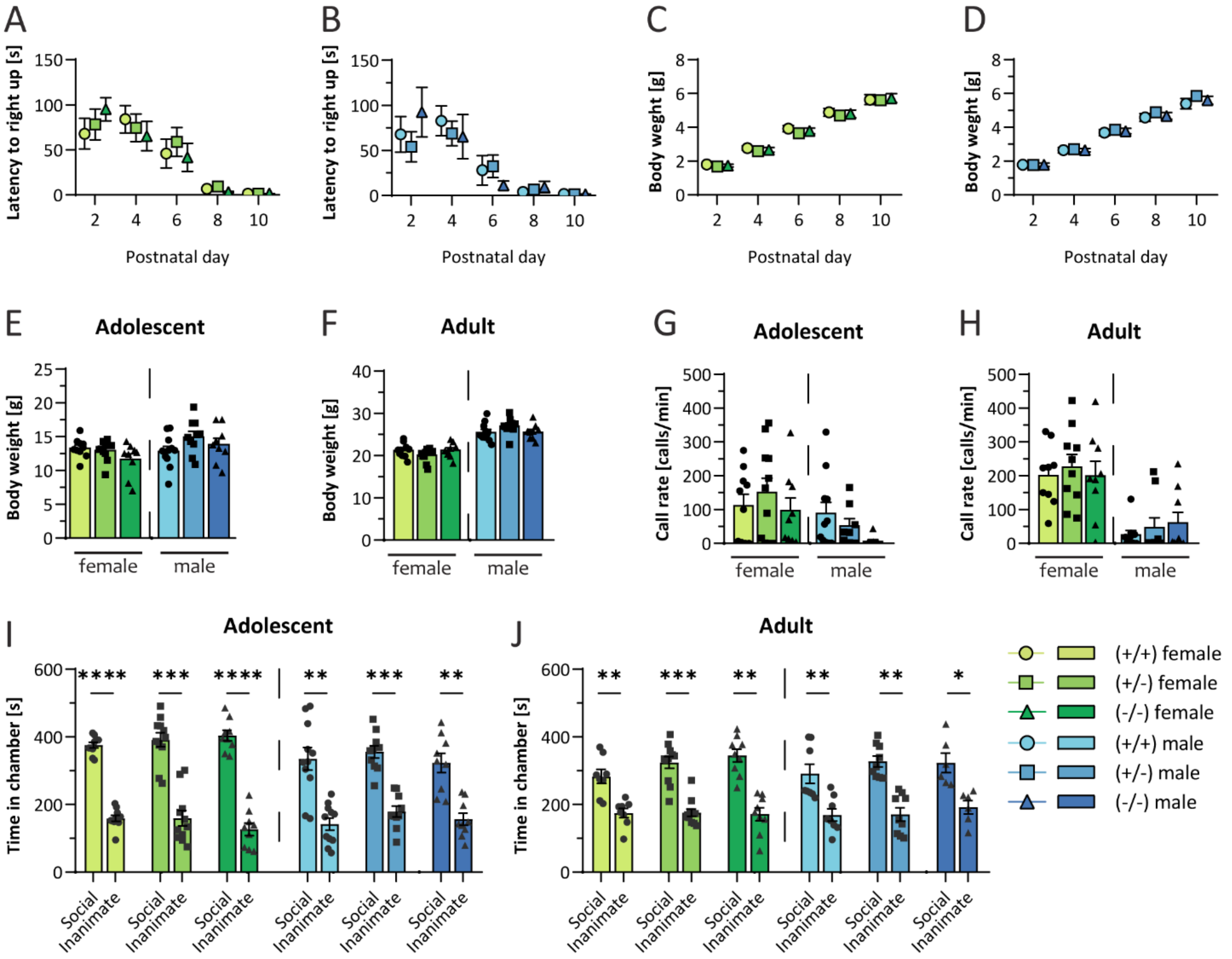

Supplementary Figure 1: (**A–B**) The righting reflex of pups and (**C–D**) the body weight were determined at postnatal day (PND) 2, PND4, PND6, PND8, and PND10 in female and male mice, respectively. Comparison of the body weight at postnatal week 4 (**E**) and postnatal week 13 (**F**) in male and female WT, *Shank3*(+/−), and *Shank3*(−/−) mice. (**G–H**) Call rate emitted during a 3 min USV recording on the direct social interaction with a same sex- and age-matched C57BL/6 stranger mouse. (**I–J**) Time spent either in the chamber with a social stimulus (same-sex and -age, stranger mouse) or in the chamber with an inanimate object in the three-chamber sociability test. All data are represented as means with SEM with 6–11 animals per group. Significance levels (p-values) were set to 0.05 ($p \leq 0.05$*, $p \leq 0.01$**, $p \leq 0.001$***, $p \leq 0.0001$****) with 95% confidence interval. See Supplementary Table 6 for detailed statistical information.

4 Supplementary Tables

Supplementary Table 1: Cohorts, age of animals, and timely sequence of behavior testing

	Female			Male			
	(+/+)	(+/-)	(-/-)	(+/+)	(+/-)	(-/-)	Age at testing
Righting reflex	10	11	9	8	10	6	P2, P4, P6, P8, P10
Body weight	10	11	9	8	10	6	P2, P4, P6, P8, P10

	Female						Male							
	(+/+)		(+/-)		(-/-)		(+/+)		(+/-)		(-/-)		Age at testing	
	1. set	2. set	1. set	2. set	1. set	2. set	1. set	2. set	1. set	2. set	1. set	2. set	1. set	2. set
Body weight	10	10	11	11	9	9	11	11	10	10	9	9	P26-P29	P89-P95
Grid hanging test	10	10	11	11	9	9	11	11	10	10	9	9	P26-P29	P89-P95
Grip strength test	10	10	11	11	9	9	11	11	10	10	9	9	P27-P30	P90-P93
Marbles burying test	10	10	11	11	9	9	11	11	10	10	9	9	P28-P31	P90-P94
Open field test	10	10	11	11	9	9	11	11	10	10	9	9	P29-P32	P91-P95
3 chamber sociability test	10	8*	11	11	9	9	11	11	10	9*	9	9	P33-P36	P92-P99
Nestlet shredding test	10	10	11	11	9	9	11	11	10	10	9	9	P35-P38	P92-P103
Self-directed behavior (self-grooming, digging)	10	10	11	11	9	9	11	11	10	10	9	9	P36-P41	P96-P104
Direct social dyadic test	10	10	11	10*	9	9	11	11	9*	10	9	9	P36-P41	P96-P104
Rotarod	10	10	11	11	9	9	11	11	10	10	9	9	P40-P47	P99-P110
Barnes Maze and reversal Barnes Maze test	10	10	11	11	9	9	11	11	10	10	9	9	P47-P62	P104-P123

*Missing animals due to technical issues with video recording software.

Supplementary Table 2: Statistical information on Figure 1

									Multiple comparison								
			Genotype		Sex		Genotype × sex		Female			Male			Pooled sexes		
		Normal							(+/+) vs	(+/+) vs	(+/-) vs	(+/+) vs	(+/+) vs	(+/-) vs	(+/+) vs	(+/+) vs	(+/-) vs
**Muscle function**	**Test**	**distributed?**	**F**	**p-value**	**F**	**p-value**	**F**	**p-value**	**(+/-)**	**(-/-)**	**(-/-)**	**(+/-)**	**(-/-)**	**(-/-)**	**(+/-)**	**(-/-)**	**(-/-)**
Grip strength, adolescent	2way ANOVA	yes	0.9074	0.4096	0.2666	0.6077	1.874	0.1633	0.9946	0.9163	0.9482	0.7811	0.0648	0.2508	0.9139	0.3897	0.6224
Grip strength, adult	2way ANOVA	yes	0.7954	0.4566	30.96	**<0.0001**	0.2257	0.7987	0.5793	0.9807	0.7162	0.7265	0.6472	0.9882	0.4305	0.7297	0.8984
Grid hanging time, adolescent	2way ANOVA	no	18.84	**<0.0001**	2.147	0.1487	1.922	0.1563	0.2767	**0.0063**	0.1876	0.6626	**<0.0001**	**0.0002**	0.2134	**<0.0001**	**0.0002**
Grid hanging time, adult	2way ANOVA	no	13.07	**<0.0001**	17.39	**0.0001**	0.9720	0.3848	0.7367	**0.0270**	0.1225	0.9960	**0.0005**	**0.0008**	0.8267	**<0.0001**	**0.0003**
Distance moved in OF, adolescent	2way ANOVA	yes	8.149	**0.0008**	3.056	0.0861	1.597	0.2118	0.3528	**0.0006**	**0.0230**	0.5653	0.2211	0.7804	0.2118	**0.0005**	0.0527
Distance moved in OF, adult	2way ANOVA	yes	11.87	**<0.0001**	1.135	0.2914	1.893	0.1604	0.6006	**0.0139**	**0.0004**	0.4443	**0.0068**	0.2011	0.9848	**0.0002**	**0.0003**

			Trial		Genotype		Trial × genotype		Multiple comparison		
**Rotarod**	**Test**	**Normal distributed?**	**F**	**p-value**	**F**	**p-value**	**F**	**p-value**	**(+/+) vs (+/-)**	**(+/+) vs (-/-)**	**(+/-) vs (-/-)**
Female, adolescent	2way ANOVA	yes	4.928	**<0.0001**	0.2691	0.7661	0.9421	0.5529	-	-	-
Trial 1									0.9406	0.9739	0.8077

Rotarod	Test	Normal distributed?	Trial		Genotype		Trial × genotype		Multiple comparison		
			**F**	**p-value**	**F**	**p-value**	**F**	**p-value**	**(+/+) vs (+/-)**	**(+/+) vs (-/-)**	**(+/-) vs (-/-)**
Trial 2									0.1491	0.7303	0.3385
Trial 3									0.5803	0.9995	0.5869
Trial 4									0.9922	0.4074	0.2983
Trial 5									0.7157	0.7667	0.9911
Trial 6									0.9572	0.6472	0.7897
Trial 7									0.7177	0.4546	0.0890
Trial 8									0.6076	0.5315	0.9677
Trial 9									0.8260	0.9962	0.7608
Trial 10									0.9625	0.9460	0.7810
Trial 11									0.6320	0.8405	0.3082
Trial 12									0.4253	0.4093	0.9864
Trial 13									0.8545	0.8837	0.5201
Trial 14									0.9796	0.9703	0.9977
Trial 15									0.9119	0.9944	0.8508
Male, adolescent	2way ANOVA	yes	7.056	**<0.0001**	7.986	**0.0019**	0.8607	0.6732	-	-	-
Trial 1									0.4257	0.1755	0.7687
Trial 2									0.6133	0.0998	0.3249
Trial 3									0.2127	**0.0270**	0.3265
Trial 4									0.2642	**0.0275**	0.5184
Trial 5									0.9703	**0.0027**	**0.0007**
Trial 6									0.9787	0.3200	0.1346
Trial 7									0.8996	0.0872	0.1437
Trial 8									0.9591	**0.0159**	**0.0352**
Trial 9									0.1368	**0.0011**	0.1654
Trial 10									0.9944	0.1240	**0.0280**
Trial 11									0.1389	0.0180	0.4350
Trial 12									0.7301	0.1046	0.3129
Trial 13									0.8545	**0.0377**	0.2392
Trial 14									0.8345	0.4728	0.7272
Trial 15									0.9938	**0.0364**	**0.0077**
Female, adult	2way ANOVA	yes	5.080	**<0.0001**	1.397	0.2646	0.6496	0.9164			
Trial 1									0.2207	0.0967	0.8938

			Trial		Genotype		Trial × genotype		Multiple comparison		
**Rotarod**	**Test**	**Normal distributed?**	**F**	**p-value**	**F**	**p-value**	**F**	**p-value**	**(+/+) vs (+/-)**	**(+/+) vs (-/-)**	**(+/-) vs (-/-)**
Trial 2									0.5648	0.9685	0.4539
Trial 3									0.8189	0.6685	0.2115
Trial 4									0.8678	0.8501	0.5456
Trial 5									0.6386	0.9329	0.8283
Trial 6									0.7307	0.6203	0.2698
Trial 7									0.4161	0.8022	0.3646
Trial 8									0.3464	>0.9999	0.3557
Trial 9									0.1838	0.3319	0.7967
Trial 10									0.9917	0.6131	0.7745
Trial 11									0.3368	0.7937	0.7315
Trial 12									0.5502	0.5941	0.9877
Trial 13									0.7313	0.7191	0.3793
Trial 14									0.7339	0.9066	0.9585
Trial 15									0.4703	0.8450	0.8485
Male, adult	2way ANOVA	yes	5.448	**<0.0001**	2.901	0.0722	0.6895	0.8830	-	-	-
Trial 1									0.8147	0.1048	0.2887
Trial 2									0.9993	0.3948	0.4106
Trial 3									0.5087	0.1048	0.3464
Trial 4									0.8332	0.4024	0.6121
Trial 5									0.6193	0.6195	0.0812
Trial 6									0.4172	0.1118	0.4107
Trial 7									0.6189	0.0543	0.7141
Trial 8									0.5097	0.1359	0.5456
Trial 9									0.4917	0.2294	0.8859
Trial 10									0.3085	0.0968	0.5328
Trial 11									0.8348	0.3045	0.3846
Trial 12									0.7064	0.1216	0.3909
Trial 13									0.6570	0.0673	0.1902
Trial 14									0.5752	0.8729	0.2352
Trial 15									0.8310	0.4063	0.5437

Supplementary Table 3: Statistical information on Figure 2

			**Multiple comparison**					
			**Female**			**Male**		
**Direct social dyadic test**	**Test**	**p- value**	**(+/+) vs (+/-)**	**(+/+) vs (-/-)**	**(+/-) vs (-/-)**	**(+/+) vs (+/-)**	**(+/+) vs (-/-)**	**(+/-) vs (-/-)**
First contact initiation, female, adolescent	Fisher's exact test	0.0045	0.1291	**0.0092**	0.2184	-	-	-
First contact initiation, male, adolescent	Fisher's exact test	0.0245	-	-	-	1.0000	**0.0422**	0.1235
First contact initiation, female, adult	Fisher's exact test	0.8849	1.0000	1.0000	1.0000	-	-	-
First contact initiation, male, adult	Fisher's exact test	0.08267	-	-	-	0.6594	0.2093	0.2547

									**Multiple comparison**					
			**Genotype**		**Sex**		**Genotype × sex**		**Female**			**Male**		
			**F**	**p-value**	**F**	**p-value**	**F**	**p-value**	**(+/+) vs**	**(+/+) vs**	**(+/-) vs**	**(+/+) vs**	**(+/+) vs**	**(+/-) vs**
**Direct social dyadic test**	**Test**	**Normal distributed?**							**(+/-)**	**(-/-)**	**(-/-)**	**(+/-)**	**(-/-)**	**(-/-)**
Time in proximity, adolescent	2way ANOVA	yes	0.3594	0.6998	3.023	0.0879	1.071	0.3498	0.9946	0.8929	0.8417	0.7157	0.2522	0.7136
Time in proximity, adult	2way ANOVA	yes	0.1044	0.9011	8.786	**0.0045**	0.4011	0.6716	0.8930	0.9984	0.9206	0.7020	0.7602	0.9971

									**Multiple comparison**								
			**Genotype**		**Sex**		**Genotype × sex**		**Female**			**Male**			**Pooled sexes**		
			**F**	**p-value**	**F**	**p-value**	**F**	**p-value**	**(+/+) vs**	**(+/+) vs**	**(+/-) vs**	**(+/+) vs**	**(+/+) vs**	**(+/-) vs**	**(+/+) vs**	**(+/+) vs**	**(+/-) vs**
**Behavior**	**Test**	**Normal distributed?**							**(+/-)**	**(-/-)**	**(-/-)**	**(+/-)**	**(-/-)**	**(-/-)**	**(+/-)**	**(-/-)**	**(-/-)**
Self-grooming, adolescent	2way ANOVA	no	17.36	**<0.0001**	0.07340	0.7875	1.187	0.3130	0.7546	**0.0130**	0.0624	0.2817	**<0.0001**	**0.0018**	0.2569	**<0.0001**	**0.0003**
Self-grooming, adult	2way ANOVA	no	14.17	**<0.0001**	0.5587	0.4580	0.3670	0.6945	0.9902	**0.0108**	**0.0129**	0.4724	**0.0004**	**0.0137**	0.6261	**<0.0001**	**0.0003**
Digging, adolescent	2way ANOVA	yes	13.94	**<0.0001**	6.212	**0.0158**	2.989	0.0587	0.5629	0.1039	0.5026	0.4404	**<0.0001**	**0.0006**	0.2557	**<0.0001**	**0.0017**
Digging adult	2way ANOVA	yes	14.66	**<0.0001**	1.094	0.3003	1.704	0.1915	0.2874	**0.0061**	0.1774	0.9360	**0.0006**	**0.0003**	0.6841	**<0.0001**	**0.0002**
Nestlet shredding, adolescent	2way ANOVA	yes	2.528	0.0892	2.139	0.1494	1.345	0.2690	0.8618	0.8989	0.9983	0.4379	**0.0215**	0.2897	0.4344	0.0734	0.5505
Nestlet shredding, adult	2way ANOVA	no	3.807	0.0284	1.196	0.2790	0.2406	0.7870	0.9746	0.1089	0.1089	0.6740	0.1980	0.6431	0.7327	0.0253	0.1306

Supplementary Table 4: Statistical information on Figure 3

									**Multiple comparison**								
			**Genotype**		**Sex**		**Genotype × sex**		**Female**			**Male**			**Pooled sexes**		
**Open Field**	**Test**	**Normal distributed?**	**F**	**p-value**	**F**	**p-value**	**F**	**p-value**	**(+/+) vs (+/-)**	**(+/+) vs (-/-)**	**(+/-) vs (-/-)**	**(+/+) vs (+/-)**	**(+/+) vs (-/-)**	**(+/-) vs (-/-)**	**(+/+) vs (+/-)**	**(+/+) vs (-/-)**	**(+/-) vs (-/-)**
Time in the border, adolescent	2way ANOVA	yes	0.0422	0.9587	1.901	0.1737	1.157	0.3221	0.6810	0.7394	0.9983	0.6077	0.4788	0.9710	0.9997	0.9890	0.9958
Time in border, adult	2way ANOVA	yes	5.527	**0.0065**	0.3324	0.5667	2.144	0.1270	0.5291	0.1188	**0.0083**	0.2507	0.0586	0.7287	0.9265	**0.0091**	**0.0236**
Time in the center, adolescent	2way ANOVA	yes	0.0075	0.9925	0.6373	0.4282	0.4239	0.6566	0.9017	0.7909	0.9670	0.9670	0.9670	0.9263	0.9927	0.9998	0.9952
Time in the center, adult	2way ANOVA	yes	5.060	**0.0097**	0.0379	0.8463	1.358	0.2658	0.6063	0.2776	**0.0392**	0.3644	**0.0324**	0.4391	0.9547	0.0140	0.0288
Entries into the center, adolescent	2way ANOVA	yes	6.203	**0.0038**	3.744	0.0583	0.2675	0.7663	0.7197	**0.0151**	0.0804	0.8391	0.1340	0.3622	0.6127	**0.0033**	**0.0386**
Entries into the center, adult	2way ANOVA	yes	15.57	**<0.0001**	0.0404	0.8413	2.978	0.0593	0.2598	**0.0102**	**<0.0001**	0.1933	**0.0006**	0.0770	0.9920	**<0.0001**	**<0.0001**
**Marbles burying test**																	
Buried marbles, adolescent	2way ANOVA	no	4.870	**0.0114**	0.03509	0.8521	0.01691	0.9832	0.7241	0.3916	0.1006	0.6108	0.3368	0.0650	0.4493	0.1383	**0.0084**
Buried marbles, adult	2way ANOVA	no	23.91	**<0.0001**	2.184	0.1453	1.056	0.3549	**0.0496**	**0.0006**	0.2032	0.0724	**<0.0001**	**0.0023**	**0.0049**	**<0.0001**	**0.0013**

**Barnes Maze test**		**Normal**	**Trial**		**Genotype**		**Trial × genotype**		**Multiple comparison**		
**Latency to escape box**	**Test**	**distributed?**	**F**	**p-value**	**F**	**p-value**	**F**	**p-value**	**(+/+) vs (+/-)**	**(+/+) vs (-/-)**	**(+/-) vs (-/-)**
Female, adolescent	Mixed-effects model (REML)	yes	15.53	**<0.0001**	4.435	0.0216	0.7495	0.7575<	-	-	-
Trial 1									0.8052	0.9505	0.5634
Trial 2									0.4950	0.2696	**0.0232**
Trial 3									0.1224	0.9959	0.1337
Trial 4									0.3617	0.8776	0.1999
Trial 5									0.1029	0.9603	0.0926
Trial 6									0.7539	0.8761	0.4373
Trial 7									0.2573	0.3169	**0.0218**
Trial 8									0.3519	0.8753	0.1411
Trial 9									0.2355	0.9072	0.1795
Trial 10									0.1924	0.6980	0.5734
Male, adolescent	Mixed-effects model (REML)	yes	31.02	**<0.0001**	1.088	0.3513	0.9541	0.5139	-	-	-
Trial 1									0.6226	0.4947	0.1849
Trial 2									0.9514	0.3796	0.2059
Trial 3									0.8623	0.7267	0.3888
Trial 4									0.5999	0.4606	0.1576
Trial 5									0.4547	0.9515	0.7374
Trial 6									0.9944	0.6781	0.8112
Trial 7									0.1735	0.9529	0.5090

**Barnes Maze test**		**Normal**	**Trial**		**Genotype**		**Trial × genotype**		**Multiple comparison**		
**Latency to escape box**	**Test**	**distributed?**	**F**	**p-value**	**F**	**p-value**	**F**	**p-value**	**(+/+) vs (+/-)**	**(+/+) vs (-/-)**	**(+/-) vs (-/-)**
Trial 8									0.6630	0.6490	0.9745
Trial 9									0.7279	0.7565	0.9964
Trial 10									0.5245	0.9677	0.7504
Female, adult	Mixed-effects model (REML)	no	12.61	**<0.0001**	12.61	0.1311	1.717	**0.0371**			
Trial 1									0.7576	**0.0307**	**0.0042**
Trial 2									0.9822	0.1384	0.0998
Trial 3									0.4961	0.8979	0.3606
Trial 4									0.6821	0.4380	0.8005
Trial 5									0.6593	0.6553	0.2949
Trial 6									0.9427	0.5224	0.6191
Trial 7									0.9997	0.6447	0.6168
Trial 8									0.9997	0.8869	0.8930
Trial 9									0.7391	0.9801	0.8288
Trial 10									0.7622	0.8401	0.9657
Male, adult	Mixed-effects model (REML)	no	8.320	**0.0002**	4.505	**0.0205**	0.5220	0.9465			
Trial 1									0.7374	0.4212	0.1640
Trial 2									0.9006	0.3197	0.6188
Trial 3									0.9017	0.7195	0.5469
Trial 4									0.2714	0.2837	0.6169
Trial 5									0.3996	0.2164	0.4835
Trial 6									0.6926	0.1807	0.1210
Trial 7									0.0787	0.5588	0.1889
Trial 8									0.8165	0.2780	0.3973
Trial 9									0.3654	0.1109	0.2564
Trial 10									0.3653	0.1188	0.2748

**Reversal Barnes Maze test**		**Normal**	**Trial**		**Genotype**		**Trial × genotype**		**Multiple comparison**		
**Latency to escape box**	**Test**	**distributed?**	**F**	**p-value**	**F**	**p-value**	**F**	**p-value**	**(+/+) vs (+/-)**	**(+/+) vs (-/-)**	**(+/-) vs (-/-)**
Female, adolescent	Mixed-effects model (REML)	yes	7.488	**<0.0001**	1.102	0.3467	0.8593	0.5730	-	-	-
Trial 1									0.9178	0.6690	0.3855
Trial 2									0.3093	0.9893	0.5019
Trial 3									0.2935	0.9900	0.3684

**Reversal Barnes Maze test**		**Normal**	**Trial**		**Genotype**		**Trial × genotype**		**Multiple comparison**		
**Latency to escape box**	**Test**	**distributed?**	**F**	**p-value**	**F**	**p-value**	**F**	**p-value**	**(+/+) vs (+/-)**	**(+/+) vs (-/-)**	**(+/-) vs (-/-)**
Trial 4									0.9974	0.8530	0.6516
Trial 5									0.9330	0.8257	0.5688
Trial 6									0.7047	0.8463	0.4710
Male, adolescent	Mixed-effects model (REML)	yes	17.58	**<0.0001**	0.9203	0.4106	0.8607	0.5716	-	-	-
Trial 1									0.9904	0.8482	0.9211
Trial 2									0.0884	0.7598	0.2402
Trial 3									0.8054	0.5777	0.9313
Trial 4									0.1805	0.9141	0.7083
Trial 5									0.1247	0.0878	0.8747
Trial 6									0.5615	0.9175	0.8485
Female, adult	Mixed-effects model (REML)	yes	15.37	**<0.0001**	1.695	0.2026	2.515	**0.0084**			
Trial 1									0.2758	0.7961	**0.0267**
Trial 2									>0.9999	0.6412	0.6062
Trial 3									0.4038	0.4512	0.9606
Trial 4									0.4126	0.7724	0.2367
Trial 5									0.8532	0.5398	0.1708
Trial 6									0.6532	0.9508	0.6864
Male, adult	Mixed-effects model (REML)	yes	11.84	**<0.0001**	1.532	0.2343	2.221	**0.0203**			
Trial 1									0.8792	0.0916	0.1383
Trial 2									0.5941	0.6475	0.2661
Trial 3									0.4173	0.3653	0.9987
Trial 4									0.2123	0.2744	0.7331
Trial 5									0.9479	0.6321	0.7538
Trial 6									0.6885	0.9901	0.8244

Supplementary Table 5: Statistical information on Figure 4

			Adolescent vs. adult					
			Female (+/+)	Female (+/-)	Female (-/-)	Male (+/+)	Male (+/-)	Male (-/-)
	**Test**	**Normal distributed?**	**p-value**	**p-value**	**p-value**	**p-value**	**p-value**	**p-value**
Grip strength	Paired two-tailed t-test	yes	0.9795	0.3035	0.5688	**0.0032**	**0.0161**	0.0977
Grid hang time	Wilcoxon matched-pairs signed rank test	no	0.5000	**0.0313**	0.2500	0.1875	0.1875	**0.0117**
Open field, distance moved	Paired two-tailed t-test	yes	0.9725	0.0587	0.5969	0.0987	**0.0431**	0.9736
Direct social dyadic test, time in proximity	Paired two-tailed t-test	yes	0.2284	0.0945	0.4225	0.9363	0.5795	0.5102
Time spent self-grooming	Wilcoxon matched-pairs signed rank test	no	**0.0254**	**0.0264**	0.7344	0.2334	0.1055	0.8008
Time spent digging	Paired two-tailed t-test	yes	**0.0079**	**0.0241**	0.1952	0.2103	**0.0008**	**0.0266**
Nesting test, processed nestlet	Wilcoxon matched-pairs signed rank test	no	**0.0137**	**0.0205**	**0.0039**	**0.0020**	**0.0137**	**0.0391**
Open field, entries into center zone	Paired two-tailed t-test	yes	0.6020	0.1293	**0.0326**	**0.0433**	0.1473	0.9292
Number of buried marbles	Wilcoxon matched-pairs signed rank test	no	**0.0059**	**0.0156**	0.5000	**0.0020**	**0.0039**	0.5000

Supplementary Table 6: Statistical information on Supplementary Figure 1

		Normal	PND		Genotype		PND × genotype		Multiple comparison		
**Pup development**	**Test**	**distributed?**	**F**	**p-value**	**F**	**p-value**	**F**	**p-value**	**(+/+) vs (+/-)**	**(+/+) vs (-/-)**	**(+/-) vs (-/-)**
Latency to right up, female	Mixed-effects model (REML)	yes	31.81	**<0.0001**	0.06662	0.9357	0.7006	0.6904	-	-	-
PND 2									0.9070	0.4338	0.7133
PND 4									0.8984	0.6825	0.9103
PND 6									0.8334	0.9822	0.7312
PND 8									0.8775	0.4519	0.3501
PND 10									0.9351	0.9424	0.7394
Latency to right up, male	Mixed-effects model (REML)	yes	23.62	**<0.0001**	0.1013	0.9041	0.7839	0.6181	-	-	-
PND 2									0.8564	0.7566	0.5025
PND 4									0.7953	0.8283	0.9916
PND 6									0.9741	0.6044	0.2934
PND 8									0.7467	0.7036	0.9112
PND 10									0.8119	0.8319	>0.9999
Body weight, female	Mixed-effects model (REML)	yes	1071	**<0.0001**	0.2820	0.7565	0.2868	0.9690	-	-	-
PND 2									0.5724	0.8509	0.8926
PND 4									0.5174	0.8323	0.8757

		Normal	PND		Genotype		PND × genotype		Multiple comparison		
**Pup development**	**Test**	**distributed?**	**F**	**p-value**	**F**	**p-value**	**F**	**p-value**	**(+/+) vs (+/-)**	**(+/+) vs (-/-)**	**(+/-) vs (-/-)**
PND 6									0.5112	0.8414	0.8443
PND 8									0.7951	0.9556	0.9414
PND 10									0.9958	0.9727	0.9296
Body weight, male	Mixed-effects model (REML)	yes	967.5	**<0.0001**	0.4181	0.6637	1.712	0.1081	-	-	-
PND 2									0.9944	0.9998	0.9907
PND 4									0.9568	0.9984	0.9518
PND 6									0.7663	0.9320	0.9396
PND 8									0.5593	0.9509	0.7083
PND 10									0.4425	0.8462	0.7184

									**Multiple comparison**								
			**Genotype**		**Sex**		**Genotype × sex**		**Female**			**Male**			**Pooled sexes**		
			**F**	**p-value**	**F**	**p-value**	**F**	**p-value**	**(+/+) vs (+/-)**	**(+/+) vs (-/-)**	**(+/-) vs (-/-)**	**(+/+) vs (+/-)**	**(+/+) vs (-/-)**	**(+/-) vs (-/-)**	**(+/+) vs (+/-)**	**(+/+) vs (-/-)**	**(+/-) vs (-/-)**
	**Test**	**Normal distributed?**															
Body weight, adolescent	2way ANOVA	yes	1.668	0.1981	4.926	**0.0307**	2.325	0.1075	0.9368	0.2316	0.3700	0.0777	0.5663	0.5143	0.3889	0.8861	0.1997
Body weight, adult	2way ANOVA	yes	0.0718	0.9308	121.0	**<0.0001**	3.235	**0.0471**	0.3909	0.9981	0.3782	0.1685	0.9983	0.2188	0.9298	0.9964	0.9611
**Direct social dyadic test**																	
USV call rate, adolescent	2way ANOVA	no	0.8530	0.4320	7.166	**0.0099**	1.463	0.2409	0.6558	0.9471	0.4729	0.7126	0.1717	0.6209	-	-	-
USV call rate, adult	2way ANOVA	no	0.2551	0.7758	42.41	**<0.0001**	0.3091	0.7355	0.8251	>0.9999	0.8201	0.8829	0.6944	0.9410	-	-	-

**3 chamber sociability test**			**Female (+/+)**	**Female (+/-)**	**Female (-/-)**	**Male (+/+)**	**Male (+/-)**	**Male (-/-)**
Time in chamber with social or inanimate stimulus	**Test**	**Normal distributed?**	**p-value**	**p-value**	**p-value**	**p-value**	**p-value**	**p-value**
Adolescent	Paired two-tailed t-test	yes	**<0.0001**	**0.0009**	**<0.0001**	**0.0011**	**0.0005**	**0.0021**
Adult	Paired two-tailed t-test	yes	**0.0095**	**0.0001**	**0.0015**	**0.0033**	**0.0018**	**0.0374**
